# Breast Cancer Survivorship, Quality of Life, and Late Toxicities

**DOI:** 10.3389/fonc.2020.00864

**Published:** 2020-06-16

**Authors:** Simone Nardin, Edoardo Mora, Feba Mariam Varughese, Francesca D'Avanzo, Ajay Ram Vachanaram, Valentina Rossi, Chiara Saggia, Sara Rubinelli, Alessandra Gennari

**Affiliations:** ^1^Department of Translational Medicine, University of Eastern Piedmont, DIMET, Novara, Italy; ^2^Center for Translational Research on Autoimmune & Allergic Disease - CAAD, Novara, Italy; ^3^Division of Oncology, Ospedale Maggiore della Carità, Novara, Italy

**Keywords:** adjuvant therapy (AT), osteoporosis, cardiotoxicity, fertility, lifestyle

## Abstract

Breast cancer is the most frequent cancer in women: in 2018, almost two million cases have been diagnosed all over the world and it represents the principal cause of death from a neoplastic disease in women. In the past years, breast cancer prognosis has significantly improved over time: currently 5-year survival rates are in the range of 90%, and 10-year survival is about 80%. This improvement has been mostly observed in western countries, due to high coverage and compliance with screening programs, leading to early diagnosis, i.e., when the disease is at a subclinical level, and to an improvement in tumor molecular characterization and innovative systemic treatments. Yet the identification of different biological breast cancer subtypes prompted the development of innovative targeted agents and improved treatment personalization. On the other hand, longer survival rates and increasing proportions of cured patients require dedicated strategies to manage long-term sequelae of breast cancer treatments, with particular attention to quality of life. This review analyzes the most important issues, potentially occurring with cancer treatments, concerning long-term sequelae and quality of life, to define a global approach to breast cancer survivorship.

## Background

It has been estimated that in the twenty-first century, cancer will be the most frequent cause of death in western countries: in fact, according to the 2015 World Health Organization (WHO) statement, cancer accounts for the most important cause of death before the age of 70, in a great proportion of countries (1).

**Figure 1:**
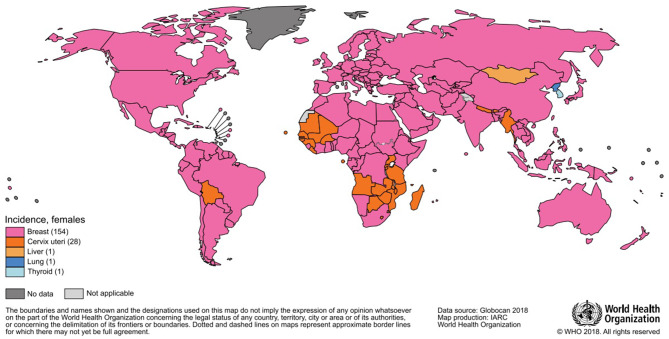
Most common type of cancer incidence in 2018 among women.

**Figure 2:**
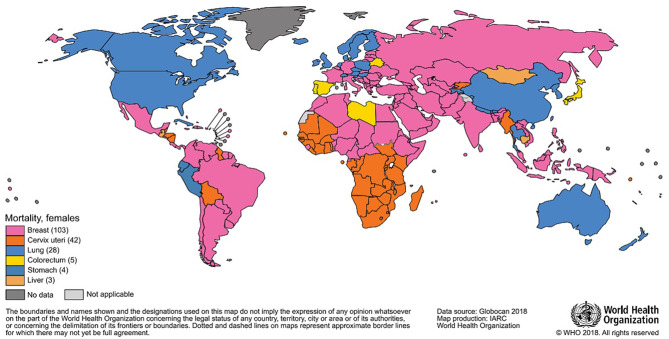
Most common type of cancer mortality in 2018 among women.

The most frequently diagnosed neoplasm is lung cancer (12% overall), and it also represents the first cause of death by cancer (18%); in women, breast cancer is the most diagnosed one and the most represented cause of death. In 2018, almost two million breast cancer cases have been diagnosed in women (Figures 1, 2), with one out of four cancer cases due to breast cancer (2).

In 2019, in Italy, 53,000 new cases of breast cancer have been reported, representing the most commonly diagnosed cancer in women: it can be estimated that about one in three cancer cases in women is represented by breast cancer. Overall, there are more than 800,000 women who have been diagnosed with breast cancer, accounting for 44% of all women living with a previous breast cancer diagnosis.

Even in 2016, breast cancer represented the major oncological reason of death in women, with more than 12,000 deaths (3). It also is the primary cause of mortality among all ages, 28% before 50 years, 21% between 50 and 69 years, and 14% after 70 years. The overall 5-year survival in Italy is in the range of 87%, without significant differences by age or region; 10-year overall survival is about 80% (4).

The recent improvements in breast cancer prognosis are mainly due to two distinct factors:

(1) Early diagnosis, i.e., when the disease is at a subclinical level, and (2) improvements in treatment personalization. The availability of large-scale population screening programs, by routine mammography, produced a significant reduction in breast cancer–related mortality in western countries; a decline in breast cancer mortality, together with an increased incidence, was recently noticed in Italy as well (5). Even if efficacy of screening mammography is still debated, usefulness of structured population programs was demonstrated by several studies (5, 6), such as the importance of an early-stage diagnosis (7). Parallel to the increased compliance to screening programs, innovative technological assays allowed an improved subtype classification that allowed innovative targeted therapies, in endocrine-sensitive and HER2-positive diseases.

It has been estimated that in 2014, 14,000 metastatic breast cancers were diagnosed in Italy, accounting for an actual prevalence in the range of 37,000 cases (8). Today, the median overall survival in patients with HER2-positive metastatic breast cancer is the range of 50 months (9–11); the recent introduction of CDK 4/6 inhibitors in the clinic also significantly improved survival in ER+ patients (12, 13). These data indicate that even in the case of metastatic disease, in a large proportion of women, breast cancer may be considered a chronic disease with an increasing proportion of long-term survivors, which can be estimated in the range of 70%.

As a consequence, there is an increasing need to develop strategies to manage breast cancer survivors with dedicated resources.

In particular, there is an urgent need to systematically approach the possible sequelae of medical treatments emerging with longer follow-up.

These include late occurring toxicities, such as chemotherapy-induced toxicity, fertility preservation in pre-menopausal women, endocrine-related bone health, and quality of life. Finally and not strictly related to toxicity sequelae or quality of life, a global approach to lifestyle interventions in breast cancer survivors, should be implemented.

## Cardiotoxicity After Adjuvant Therapy

Nowadays, breast cancer prognosis, whether early or advanced setting, has improved noticeably. This is in part due to the availability and large-scale use of new treatment solutions (14). However, some of these agents may cause short-term and long-term side effects that can sometime be life threatening. One of the most important side effects of breast cancer adjuvant treatments is cardiac toxicity. In particular, late cardiotoxicity might occur years after the administration of adjuvant therapies and is mainly related to the use of adjuvant anthracyclines and trastuzumab; endocrine therapy (12) and chest wall radiotherapy, especially when left breast is involved, can also have an impact on cardiac toxicity. Cardiotoxicity is mainly due to a direct effect on cardiomyocytes, leading to cell death and permanent or transient left ventricular ejection fraction reduction, resulting in symptomatic congestive heart failure in some cases (15). It is noteworthy to remember that cardiotoxicity induced by breast cancer treatments also includes vascular disorders, arrhythmias, and ischemia (16, 17).

For these reasons, the assessment of baseline risk of potential cardiotoxicity is really crucial before starting treatment. It is important to investigate and check the existence of risk factors related to lifestyle habits (smoking, alcohol intake, obesity, sedentary attitude), demographic features (age, family history, hypertension, diabetes mellitus, hypercholesterolemia), previous cardiotoxic therapy, or any event related to heart disease (heart failure, asymptomatic left ventricular systolic dysfunction, cardiomyopathy, or coronary artery disease). In fact, their presence can increase the risk of symptomatic cardiac dysfunction (18).

Heart examinations during follow-up are recommended at definite intervals after adjuvant anthracyclines and trastuzumab, although no clear indication on how long this approach should be maintained is provided.

## Ovarian Failure and Fertility Preservation

Among mid-term toxicities with a strong impact on quality of life, fertility impairment is one of the most important factors in younger patients who are candidates for systemic adjuvant therapies. This issue is of particular importance due to the substantial increase in the incidence of breast cancer in European women in their 20s and 30s (19). According to individual disease characteristics, a relevant proportion of these young women will require adjuvant treatments, including chemotherapy, and will receive drugs associated with different magnitudes of gonadotoxicity. Increased risk of premature ovarian failure is mainly related to the use of alkylating agents such as cyclophosphamide, while anthracyclines and taxanes have shown an intermediate risk. Methotrexate and 5-fluorouracil have been associated with a low risk of ovarian damage (20). As a consequence, fertility preservation techniques should be discussed with all young women requiring adjuvant chemotherapy. In this setting, the following options are available: oocyte cryopreservation, embryo cryopreservation, ovarian tissue cryopreservation, and ovarian suppression mediated by gonadotropin releasing hormone analogs (GnRHa).

Cryopreservation of embryos and oocytes is considered the standard approach and is currently recommended by consensus of experts and international literature (21). In the past years, embryo cryopreservation represented the most widely approved procedure for fertility preservation; however, since 2013, cryopreservation of oocytes is no longer considered experimental and is currently recommended to the majority of young women (22). The most important benefit of oocyte cryopreservation over embryo cryopreservation is the potential use in patients without a partner and feasibility in countries where embryo cryopreservation is not allowed.

Ovarian tissue cryopreservation is an experimental method of freezing and transplantation. Ovarian tissue can be stored as entire ovary, fragments of ovarian cortex, or isolated follicles; however, when the tissue is re-implanted, concerns have been raised on the potential hypoxia-induced damage, leading to loss of primordial follicles and increased risk of implanting malignant cells (23).

Finally, the concomitant administration of GnRHa has been reported to reduce gonadal toxicity mediated by chemotherapy (24). Recently, a systematic review conducted on individual patient data evaluated the efficacy of this approach in patients affected by early breast cancer. In this study, 873 patients were included from five clinical trials comparing adjuvant chemotherapy with adjuvant chemotherapy plus synthetic GnRHa. Among the 873 patients, 37 (10.3%) women had at least one post-treatment pregnancy in the GnRHa group vs. 20 (5.5%) in the control group (HR, 1.83; 95% CI, 1.06–3.15; *P* = 0.03). No significant differences in disease-free survival and overall survival were observed. These data confirmed the efficacy and safety of temporary ovarian suppression with GnRHa during chemotherapy. After these data, ovarian function suppression achieved by the administration of GnRHa during adjuvant chemotherapy in fertile women currently represents the most prescribed approach to decrease the likelihood of chemotherapy-induced premature ovarian failure and preserve fertility in premenopausal women (25).

## Distress, Body Image, Self-Esteem, and Sexuality

Breast cancer experience is often associated with relevant physical and psychosocial changes in affected women. The impact of breast cancer diagnosis is different across the lifespan, since younger patients have increased risk of depression, anxiety, and intrusive thoughts (26). High degree of psychosocial adaptation, family relationship, and support networks represent protective factors for distress, because of their role in counteracting experience of loneliness and sense of isolation. The experience of anxiety and fear of the future is common in 20–30% of patients, in a metaphorical “sword of Damocles,” related to the perceived risk of disease recurrence and death. Distress due to the disruption of body image in breast cancer, linked to hair loss, paleness, weight gain, and discontent for aesthetical outcomes of surgery, is also reported (27).

In this perspective, hospital-based programs of beauty care intervention can have beneficial effect in patients with breast cancer. A group makeup workshop is a low-cost intervention with patient-reported outcomes of distress reduction and amelioration of quality of life; moreover, this approach has been shown to immediately build confidence and self-esteem, with short-term and midterm benefic effects (28).

Furthermore, a low self-esteem affects self-perceived attractiveness and consequently intimacy and sex life. Sexuality is a complex area, including psychosocial, sociocultural, and biological aspects. Satisfaction in one's sex life can be a critical issue for the quality of life in breast cancer patients and should be included in individual patient assessment. Sexual dysfunction is more frequently observed in patients with breast cancer than in healthy women, and treatment-related adverse effects can have a prolonged, social negative impact (29). With these factors in mind, sexual satisfaction in breast cancer survivors deserves more attention, particularly in pre/peri-menopausal patients (30). Sexuality should be included in assessment (alone or with partner). Additionally, women prefer being informed by professional figures (preferably nurse or primary doctor) about potential issues in sex health and related solutions (31); this should also be taken into account. In conclusion, sexual counseling can be useful to patients and to their partners, to help improve quality of life during the cancer experience.

## Osteoporosis and Long-Term Survival

Osteoporosis is by far the most common problem in terms of bone health in the aging female population in most industrialized countries. The lifetime risk of fractures among US and European women at the age of 50 is about 40% with a risk of hip fracture in the range of 15–20%.

Being an estrogen-dependent tissue, bone is strongly affected by its circulating levels. Breast cancer patients with endocrine sensitive disease are candidate to receive adjuvant endocrine therapy with aromatase inhibitors (AIs) for 5–10 years according to the individual risk of recurrence; this treatment currently represents the standard of care for postmenopausal women and for high-risk premenopausal patients. Yet, the decrease in circulating estrogen levels associated to AIs can produce a rapid increase in the potential risk of fractures (32). Data from adjuvant clinical trials do not comprehensively represent the true impact of the related increased risk of fractures, especially in women with no baseline osteoporosis. At the same time, the long-term risk for factures in premenopausal women at the time of breast cancer diagnosis is still poorly recognized (33, 34). In the clinical practice, a baseline evaluation of fracture risk in postmenopausal and premenopausal women with early disease, candidate to AIs, should be regularly performed and repeated on a 2-year basis, in the absence of bone-related symptoms or events. The adoption of pharmacologic interventions to prevent bone loss is supported by a number of randomized clinical trials showing that bisphosphonates may be active also in women with a high risk of fracture following cancer treatment. Based on these results, guidelines recommend treatment in women with a *T*-score ≤-2 or those with at least two clinical risk factors.

Recently, denosumab, an anti-RANK ligand antibody, also approved for fracture prevention in the healthy postmenopausal woman, has been shown to extend the time to first fracture in breast cancer postmenopausal women treated with AIs. These benefits have led clinicians to consider denosumab as a key therapeutic option in the prevention of AI-induced bone loss. However, several issues still need to be addressed regarding the use of these different agents in an adjuvant setting (35). It is also worth mentioning that women receiving AIs are at higher risk of developing periodontal disease, with a possible impact on quality of life (36). In our clinical setting, we have implemented a separate consultation, where all women are regularly (every year) supervised by dental hygienists: this dedicated approach was appreciated by patients, and the incidence of periodontal disease was reduced (unpublished data).

## Host Metabolism and Lifestyle In Breast Cancer Survivors

In addition to all the aspects previously described, when dealing with long-term survivorship, particular attention should be dedicated to metabolic aspects including weight control and management of physical inactivity, through lifestyle interventions. This issue, although not strictly related to long-term toxicity from adjuvant treatments or quality of life, might become the leading survivorship emergency in the short period, due to the well-known and increasingly proven interactions between altered metabolism and breast cancer prognosis (37–40). A possible approach should include innovative care strategies, non-hospital based, to overcome the risk of excessive medicalization in breast cancer survivorship.

## Conclusions

Breast cancer survivorship represents one of the most challenging aspects to be approached in dedicated clinical follow-up settings. This is mainly due to improvements in survival that have occurred over the past 20 years, leading to disease chronicization in advanced stages and cure in early stages. In this review, we have discussed the most important treatment sequelae occurring with cancer treatments that require appropriate management and dedicated resources. This aspect is particularly important since today we interrupt breast-specific follow-up 5–10 years after breast cancer diagnosis. In the perspective of ameliorating the overall quality of life of breast cancer survivors, however, additional resources must be allocated to manage “breast cancer survivorship.”

## Author Contributions

All authors contributed to the manuscript. SN, AG, FD'A, and SR wrote the manuscript. AG provided final revision.

## Conflict of Interest

The authors declare that the research was conducted in the absence of any commercial or financial relationships that could be construed as a potential conflict of interest.
